# Evaluation of the major histocompatibility complex (MHC) class II as a candidate for sudden acquired retinal degeneration syndrome (SARDS) in Dachshunds

**DOI:** 10.1111/vop.12646

**Published:** 2019-02-21

**Authors:** Stephanie J. Stromberg, Sara M. Thomasy, Ariana D. Marangakis, Soohyun Kim, Ann E. Cooper, Emily A. Brown, David J. Maggs, Danika L. Bannasch

**Affiliations:** ^1^ Department of Surgical and Radiological Sciences, School of Veterinary Medicine University of California Davis California; ^2^ Department of Population Health and Reproduction, School of Veterinary Medicine University of California Davis California; ^3^ Department of Ophthalmology & Vision Science, School of Medicine UC Davis Medical Center Sacramento California

**Keywords:** Dachshund, DLA, dog leukocyte antigen, genome‐wide association studies, MHC, sudden acquired retinal degeneration syndrome

## Abstract

**Objective:**

Sudden acquired retinal degeneration syndrome (SARDS) is one of the leading causes of acute blindness in dogs, with an unknown etiology and no effective treatment. Certain breeds such as Dachshunds are overrepresented among SARDS patients, and therefore, the syndrome is suspected to have a genetic component. The objective of this study was to determine if a genetic locus associated with SARDS in Dachshunds could be identified using a genome‐wide association study (GWAS).

**Procedures:**

Genome‐wide association mapping was performed in 15 SARDS‐affected and 16 unaffected Dachshunds. Genotyping of three classical DLA class II genes (*DLA‐DRB1, DLA‐DQA1*, and *DLA‐DQB1*) was performed in 34 SARDS‐affected and 66 unaffected Dachshunds to evaluate for an association in this region.

**Results:**

Although no single nucleotide polymorphisms (SNPs) were of genome‐wide statistical significance (*P*_Bonferroni_ < 0.05), 5 of the top 9 SNPs were in the major histocompatibility complex (MHC). Using DLA typing, the allele DLA‐DRB1*09401 was identified as a risk factor for the development of SARDS (*P* = 0.0032, OR = 4.0). The alleles DLA‐DQB1*00101 (P = 0.0050, OR = 0.31), DLA‐DQA1*00901 (*P = *0.0087, OR = 0.33), and a previously identified *DLA‐DRB1*allele described as “DRB1‐T” (*P = *0.0284, OR = 0.37) were identified as protective factors.

**Conclusions:**

Although far from definitive, association of SARDS with alleles of immunologic importance further supports the hypothesis that autoimmunity may play a role in the pathogenesis of SARDS.

## 1 INTRODUCTION

Sudden acquired retinal degeneration syndrome (SARDS) is a common cause of irreversible blindness in dogs.^1^ The disease is typically acute in onset and characterized by an initially normal fundic appearance despite an extinguished electroretinogram. Currently, the etiology of SARDS remains unknown and there is no proven treatment for the condition, although several different etiologic hypotheses exist and several therapeutic approaches have been suggested. Because dogs with SARDS often present with concurrent systemic signs such as polyuria, polydipsia, polyphagia, lethargy, and weight gain, as well as laboratory abnormalities consistent with hyperadrenocorticism, some researchers hypothesize that SARDS is an endocrine disorder.^1^ In one study, >90% of dogs with SARDS had elevated adrenal sex hormone and/or cortisol serum concentrations.^2^ By contrast, others suggest that SARDS is the canine equivalent of autoimmune retinopathy (AIR)—a disease of humans in which antiretinal antibodies result in vision loss and severe attenuation or loss of retinal electrical activity, with minimal changes in funduscopic appearance.^3^, ^4^ In one study, an autoantibody to neuron‐specific enolase (NSE)—a glycolytic enzyme found in photoreceptors, neurons, and neuroendocrine tissues—was identified in 6 of 24 dogs with SARDS, while no control dogs had detectable autoantibodies.^5^

In 2014, the American College of Veterinary Ophthalmologists' Vision for Animals Foundation hosted a think tank to prioritize goals for future SARDS research.^1^ One of their specific recommendations was to use genome‐wide association studies (GWAS) to identify genetic factors that may contribute to an understanding of the etiologic and pathogenic mechanisms of the disease. This technology uses single nucleotide polymorphisms (SNPs) at thousands of loci across the entire canine genome to identify regions of genetic difference between control and affected animals. If a statistically significant association is found between a region of the canine genome and the disease of interest, candidate genes within the region can be identified for further investigation. Identification of a genetic association would increase understanding of SARDS, refine the direction of future research, and could ultimately lead to the development of treatments and identification of at‐risk patients. Furthermore, it could support or refute the hypothesis that SARDS may serve as a spontaneous canine model for AIR.

A recent retrospective study conducted at the University of California‐Davis Veterinary Medical Teaching Hospital (UCD‐VMTH) identified several breeds that were overrepresented in the SARDS patient population.^6^ Dachshunds, in particular, were 8‐fold overrepresented, which is consistent with previous reports^7^ and may indicate a genetic component of the disease. Therefore, the purpose of the present study was to use genome‐wide association to investigate genetic associations with SARDS in Dachshunds.

## 2 MATERIALS AND METHODS

### 2.1 Animals

This study was approved by the Institutional Animal Care and Use Committee at the University of California‐Davis (Protocol #18719) and performed in accordance with the Association for Research in Vision and Ophthalmology's guidelines for the use of animals in research. Blood samples were collected from SARDS‐affected and unaffected Dachshunds at the UCD‐VMTH, as well as from animals recruited through outreach to veterinary ophthalmologists and to Dachshund breed organizations within the United States.

Inclusion criteria for SARDS‐affected Dachshunds included sudden and complete vision loss, examination and diagnosis by a board certified veterinary ophthalmologist or ophthalmology resident in training, a bilaterally extinguished electroretinogram (ERG), and lack of an explanation for sudden and complete vision loss on fundic examination (eg, total retinal separation, diffuse severe chorioretinitis). Dogs diagnosed with SARDS were excluded from the current study if they had intraocular abnormalities impeding adequate visualization of the ocular fundus. All Dachshunds in the control population were >8 years old, had an intact menace response and/or pupillary light reflexes, and were reported to be visual by their owner. Dachshunds <8 years of age were excluded in order to avoid dogs that may be genetically predisposed to SARDS but had not yet developed signs of the syndrome. Exclusion criteria for control dogs included Cushing's disease, progressive retinal atrophy, notable regions of retinal degeneration or detachment on fundic examination, and/or an abnormal ERG in one or both eyes.

With the exception of one sample that did not have enough DNA remaining after the GWAS, all DNA samples used for the GWAS were also used in major histocompatibility complex (MHC) genotyping. Additional control and SARDS‐affected dogs were recruited for MHC genotyping, and a number of control DNA samples were obtained from the Canine Genetics Biorepository (CGB), a database of gDNA samples collected from VMTH patients (Protocol # 20356). The CGB DNA samples were only used if medical record review demonstrated that they met the same inclusion criteria listed above.

### 2.2 Genome‐wide association study

Whole blood from affected and control dogs was collected into tubes containing ethylenediaminetetraacetic acid, DNA was extracted using the Puregene kit (Qiagen, CA), and DNA concentration was determined with a nanodrop spectrophotometer. DNA samples were genotyped with the Axiom™ Canine Genotyping Array Set B (Applied Biosystems™, CA). The manufacturer's quality control metrics were applied, and SNPs with less than 90% genotyping call rates and a minor allele frequency over 0.05 were removed. Samples from affected and control Dachshunds were analyzed for population stratification by quantile‐quantile (Q‐Q) plots. Bonferroni correction was utilized to account for the number of tests performed. Association analysis was performed using PLINK software (Shaun Purcell, URL: http://pngu.mgh.harvard.edu/purcell/plink/).^8^

### 2.3 MHC genotyping for DLA‐DRB1, DQA1, and DQB1

Direct sequencing of genomic DNA (gDNA) from affected and control Dachshunds was performed using locus‐specific intronic primers targeting the following 3 dog leukocyte antigen (DLA) class II genes: *DLA‐DRB1*, *DLA‐DQA1*, and *DLA‐DQB1*. Forward and reverse primers used for these loci (respectively) were DRB.F (GATCCCCCCGTCCCCACAG) and DRB.R (TGTGTCACACACCTCAGCACCA), DQA.F (TAAGGTTCTTTTCTCCCTCT) and DQA.R (GGACAGATTCAGTGAAGAGA), and DQB.F2 (GGTTGACGGGCATCAGAG) and DQB.R (GGTGCGCTCACCTCGCCGCT). Each polymerase chain reaction (PCR) was performed with gDNA in a 20‐µL reaction containing 1 × PCR buffer (Applied Biosystems, Foster City, CA), 0.8 µmol/L of each primer, 0.125 mmol/L dNTP, and 0.5 U of AmpliTaq Gold (ABI PRISM BigDye Terminator Cycle Sequencing Ready Reaction Kits, Original and Version 2.0 Protocol, Applied Biosystems). A negative control was included with each reaction to identify any reagent contamination. For amplification, samples were first held at 94°C for 12 minutes then underwent 35 cycles of 94°C for 30 seconds; 66°C (DRB), 57°C (DQA), or 60°C (DQB) for 30 seconds; and 72°C for 45 seconds, followed by a final extension phase of 72°C for 20 minutes. The final product sizes were 290 bp for *DLA‐DRB1*, 312 bp for *DLA‐DQA1*, and 372 bp for *DLA‐DQB1*. To remove unconsumed dNTPs and primers, PCR products were cleaned using the ExoSap‐IT^®^ kit (Applied Biosystems, CA) according to the manufacturer's protocol. Sequencing reactions were performed with Big Dye® terminator mix (Applied Biosystems, CA) using the aforementioned primers according to the manufacturer's protocol. The purified PCR products were sequenced on an ABI 3500 Genetic Analyzer (Applied Biosystems, CA).

### 2.4 DNA allele and haplotype assignment

DRB1/DQA1/DQB1 allele determination was performed by comparison with annotated sequences in GenBank, beginning with homozygous individuals. For heterozygotes, a comparison was made of GenBank sequences that most closely matched the sequence data, identifying the two alleles that, when combined, could explain all heterozygous sequencing calls. Three‐loci DRB1/DQA1/DQB1 haplotypes were identified in a similar manner, beginning with homozygous individuals and identifying heterozygote haplotypes by comparing to haplotypes identified in homozygous animals.^9^ *P*‐values and odds ratios (OR) with 95% confidence intervals (CI) were calculated for allele and haplotype frequencies in SARDS‐affected and control Dachshunds using a 2 × 2 contingency table. Adjustments for multiple comparisons were not performed, as previously recommended for similar studies.^10^, ^11^

## 3 RESULTS

### 3.1 Genome‐wide association study

Using the inclusion criteria and methods outlined, DNA samples were prospectively collected from 16 Dachshunds diagnosed with SARDS and 16 Dachshunds considered unaffected (controls). Of these samples, 15 affected and 16 control samples passed filters and quality control. A genomic inflation factor (λ) of 1 was observed, and 404 619 SNPs remained after quality control.

**Figure 1:**
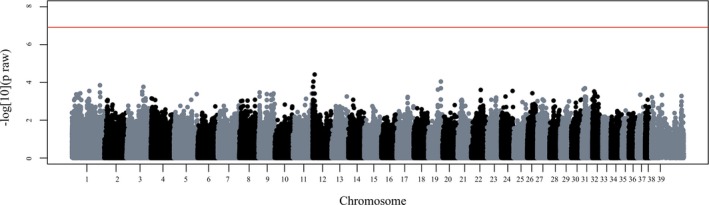
Genome‐wide association study (GWAS) of sudden acquired retinal degeneration syndrome (SARDS) in Dachshunds. Manhattan plot showing –log_10_ of the raw *P*‐values for each of 404 619 single nucleotide polymorphisms (SNPs) compared between 15 SARDS‐affected and 16 unaffected (control) Dachshunds, arranged by chromosome (x axis). Line denotes genome‐wide significance based on Bonferroni‐corrected *P*‐values. Genomic inflation factor = 1

No SNPs were of genome‐wide statistical significance (*P*_Bonferroni_ < 0.05 (Figure 1). Six of the nine most statistically significant SNPs were found on canine chromosome (CFA) 12, and five of these six were within the coding region of the major histocompatibility complex (MHC) (Table 1). Statistically significant genome‐wide associations to the MHC are uncommon in dogs; therefore, this association was investigated further by MHC genotyping a larger cohort of SARDS‐affected and control Dachshunds.

**Table 1:** Nine single nucleotide polymorphisms (SNPs) identified as most closely associated with sudden acquired retinal degeneration syndrome (SARDS) during a genome‐wide association study (GWAS) in 15 SARDS‐affected and 16 control Dachshunds

SNP	Chromosome	Position (bp)	χ^2^	*P*‐value
AX‐167952531	12	8 184 905	16.95	3.831E‐5
AX‐167543896	12	3 268 272	15.33	9.018E‐5
AX‐167539614	19	48 026 694	15.33	9.018E‐5
AX‐167921313	1	106 754 682	14.48	1.414E‐4
AX‐167811284	3	63 770 730	14.11	1.723E‐4
AX‐167798146	12	1 936 523	14.11	1.723E‐4
AX‐167837680	12	1 939 111	14.11	1.723E‐4
AX‐167809780	12	1 950 348	14.11	1.723E‐4
AX‐167698387	12	1 956 879	14.11	1.723E‐4

Base pair (bp) positions of SNPs are based on CanFam3.1 assembly. The approximate location of the major histocompatibility complex (MHC) coding region in CanFam 3.1 is CFA12: 800 000‐2 600 000 Mb.

### 3.2 MHC genotyping for DLA‐DRB1, DQA1, and DQB1

**Figure 2:**
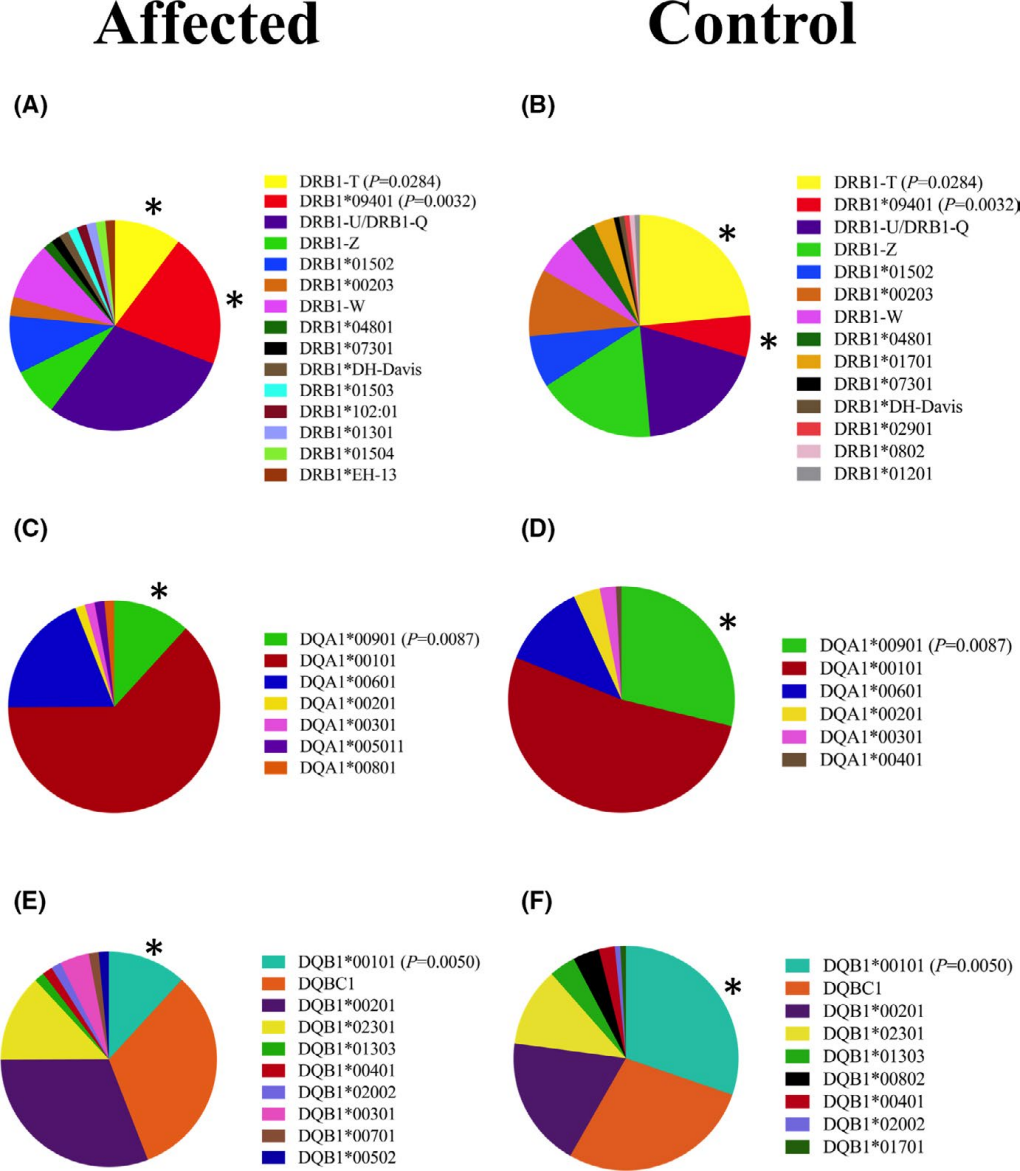
Dog leukocyte antigen (DLA) typing results for 34 Dachshunds affected with sudden acquired retinal degeneration syndrome (SARDS) and 66 unaffected (control) Dachshunds. A, *DLA‐DRB1* allele frequencies in affected dogs. B, *DLA‐DRB1* allele frequencies in control dogs. C, *DLA‐DQA1*allele frequencies in affected dogs. D, *DLA‐DQA1*allele frequencies in control dogs. E, *DLA‐DQB1*allele frequencies in affected dogs. F, *DLA‐DQB1*allele frequencies in control dogs. Statistically significant differences between affected and control dogs are indicated with an asterisk, and *P*‐values are written in the key

**Figure 3:**
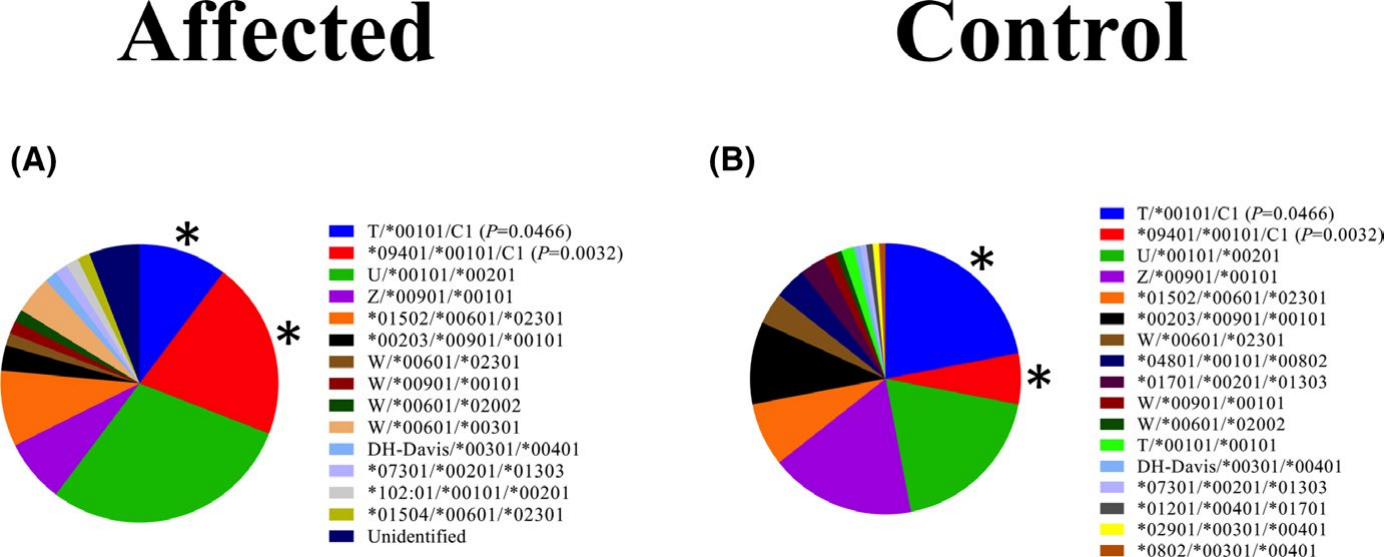
Dog leukocyte antigen (DLA) typing results for 34 Dachshunds affected with sudden acquired retinal degeneration syndrome (SARDS) and 66 unaffected (control) Dachshunds. A, haplotype frequencies in affected dogs. B, haplotype frequencies in control dogs. Statistically significant differences between affected and control dogs are indicated with an asterisk, and *P*‐values are written in the key

In all, DNA samples from 34 Dachshunds affected with SARDS and 66 control Dachshunds were typed for three DLA class II genes. Of the 66 control Dachshunds, 31 samples were taken from the CGB database. The entire study population contained 19 different *DLA‐DRB1*alleles, 8 different *DLA‐DQA1*alleles, and 12 different *DLA‐DQB1*alleles (Table 2, Figure 2), which combined into 25 different haplotypes (Table 3, Figure 3). A previously unidentified allele (hereafter “DLA‐DRB1*DH‐Davis”) was detected in 2 heterozygous dogs as part of the same novel haplotype (hereafter referred to as “DLA‐DRB1*DH‐Davis/DQA*00301/DQB*00401”).

**Table 2:** Prevalence of different alleles of three dog leukocyte antigen (DLA) class II genes (*DLA‐DRB1*, *DLA‐DQA1*, and *DLA‐DQB1*) in 34 Dachshunds with sudden acquired retinal degeneration (SARDS) and 66 unaffected (control) Dachshunds

DLA Gene/allele	SARDS‐affected dogs		Control dogs		*P*‐value	OR (95% CI)
	Number	Frequency	Number	Frequency		
*DQA1*						
*00101	43	0.63	69	0.52	0.1402	1.57 (0.86‐2.86)
*00901	**8**	**0.12**	**38**	**0.29**	**0.0087**	**0.33 (0.14‐0.76)**
*00601	13	0.19	16	0.12	0.1865	1.71 (0.77‐3.81)
*00201	1	0.01	5	0.04	0.3804	0.38 (0.04‐3.31)
*00301	1	0.01	3	0.02	0.7033	0.64 (0.06‐6.29)
*00401	0	0	1	0.01	0.7854	0.64 (0.03‐15.92)
*005011	1	0.01	0	0	0.2796	5.89 (0.24‐146.51)
*00801	1	0.01	0	0	0.2796	5.89 (0.24‐146.51)
*DQB1*						
*C1*(AH006318.2)	22	0.32	37	0.28	0.5257	1.23 (0.65‐2.32)
*00101	**8**	**0.12**	**40**	**0.30**	**0.0050**	**0.31 (0.13‐0.70)**
*00201	21	0.31	25	0.19	0.0594	1.91 (0.97‐3.75)
*02301	9	0.13	15	0.11	0.6999	1.19 (0.49‐2.88)
*01303	1	0.01	5	0.04	0.3804	0.38 (0.04‐3.31)
*00802	0	0	5	0.04	0.2314	0.17 (0.01‐3.11)
*00401	1	0.01	3	0.02	0.7033	0.64 (0.07‐6.29)
*02002	1	0.01	1	0.01	0.6373	1.96 (0.12‐31.75)
*00301	3	0.04	0	0	0.0811	14.16 (0.72‐278.23)
*00701	1	0.01	0	0	0.2796	5.89 (0.24‐146.51)
*00502	1	0.01	0	0	0.2796	5.89 (0.24‐146.51)
*01701	0	0	1	0.01	0.7854	0.64 (0.03‐15.92)
*DRB1*						
*‐U* (DQ056278.1)	20	0.29	25	0.19	0.0952	1.78 (0.90‐3.52)
***‐T* (DQ056277.1)**	**7**	**0.10**	**31**	**0.23**	**0.0284**	**0.37 (0.16‐0.90)**
*‐Z* (DQ056280.1)	5	0.07	23	0.17	0.0592	0.38 (0.14‐1.04)
*09401	**14**	**0.21**	**8**	**0.06**	**0.0032**	**4.02 (1.59‐10.14)**
*01502	6	0.09	10	0.08	0.7582	1.18 (0.41‐3.40)
*00203	2	0.03	13	0.10	0.0980	0.28 (0.06‐1.27)
*‐W* (DQ056281.1)	6	0.09	8	0.06	0.4706	1.50 (0.50‐4.51)
*04801	1	0.01	5	0.04	0.3804	0.38 (0.04‐3.31)
*01701	0	0	4	0.03	0.2952	0.21 (0.01‐3.93)
*07301	1	0.01	1	0.01	0.6373	1.96 (0.12‐31.75)
*DH‐Davis	1	0.01	1	0.01	0.6373	1.96 (0.12‐31.75)
*01503	1	0.01	0	0	0.2796	5.89 (0.24‐146.51)
*02901	0	0	1	0.01	0.7854	0.64 (0.03‐15.92)
*0802	0	0	1	0.01	0.7854	0.64 (0.03‐15.92)
*102:01	1	0.01	0	0	0.2796	5.89 (0.24‐146.51)
*01301	1	0.01	0	0	0.2796	5.89 (0.24‐146.51)
*01504	1	0.01	0	0	0.2796	5.89 (0.24‐146.51)
*01201	0	0	1	0.01	0.7854	0.64 (0.03‐15.92)
*EH‐13(EU528639.1)	1	0.01	0	0	0.2796	5.89 (0.24‐146.51)

Where GenBank sequence of alignment is not named according to standard practice, accession number is provided in parentheses. A previously unidentified *DLA‐DRB1* allele is referred to as DRB1*DH‐Davis. Alleles in bold type are those whose frequency was significantly higher or lower in SARDS‐affected animals.

CI, confidence interval; OR, odds ratio.

**Table 3:** Prevalence of dog leukocyte antigen (DLA) class II haplotypes in 34 Dachshunds with sudden acquired retinal degeneration (SARDS) and 66 unaffected (control) Dachshunds

DLA haplotype *(DLA‐DRB1/DQA1/DQB1)*	SARDS‐affected dogs			Control dogs		*P*‐value		OR (95% CI)	
	Number		Frequency	Number	Frequency				
U/*00101/*00201	20	0.29		25	0.19		0.0952		1.78 (0.90‐3.52)
T/*00101/C1	**7**	**0.10**		**29**	**0.22**		**0.0466**		**0.41 (0.17‐0.99)**
Z/*00901/*00101	5	0.07		23	0.17		0.0592		0.38 (0.14‐1.04)
*09401/*00101/C1	**14**	**0.21**		**8**	**0.06**		**0.0032**		**4.02 (1.59‐10.14)**
*01502/*00601/*02301	6	0.09		10	0.08		0.7582		1.18 (0.41‐3.40)
*00203/*00901/*00101	2	0.03		13	0.10		0.0980		0.28 (0.06‐1.27)
W/*00601/*02301	1	0.01		5	0.04		0.3804		0.38 (0.04‐3.31)
*04801/*00101/*00802	0	0		5	0.04		0.2314		0.17 (0.01‐3.11)
*01701/*00201/*01303	0	0		4	0.03		0.2952		0.21 (0.01‐3.93)
W/*00901/*00101	1	0.01		2	0.01		0.9804		0.97 (0.09‐10.89)
W/*00601/*02002	1	0.01		1	0.01		0.6373		1.96 (0.12‐31.75)
W/*00601/*00301	3	0.04		0	0		0.0811		14.16 (0.72‐278.23)
T/*00101/*00101	0	0		2	0.01		0.5353		0.38 (0.02‐8.05)
*DH‐Davis/*00301/*00401	1	0.01		1	0.01		0.6373		1.96 (0.12‐31.75)
*07301/*00201/*01303	1	0.01		1	0.01		0.6373		1.96 (0.12‐31.75)
*01201/*00401/*01701	0	0		1	0.01		0.7854		0.64 (0.03‐15.92)
*02901/*00301/*00401	0	0		1	0.01		0.7854		0.64 (0.03‐15.92)
*0802/*00301/*00401	0	0		1	0.01		0.7854		0.64 (0.03‐15.92)
*102:01/*00101/*00201	1	0.01		0	0		0.2796		5.89 (0.24‐146.51)
*01504/*00601/*02301	1	0.01		0	0		0.2796		5.89 (0.24‐146.51)
Unidentified	4	0.06		0	0		N/A		N/A

A new haplotype containing the novel DRB1*DH‐Davis allele was identified in two dogs. Haplotypes in bold type are those whose frequency was significantly higher or lower in SARDS‐affected animals.

CI, confidence interval; OR, odds ratio.

The allele frequency of DLA‐DRB1*09401was significantly higher in affected dogs (20.6%) than in control dogs (6.1%; OR = 4.0; CI = 1.6‐10.1; *P = *0.0032; Table 2; Figure 2). This allele was found in a single haplotype (DLA‐DRB1*09401/DQA*00101/DQB‐DQBC1) which therefore shared the identical frequencies as the allele alone. Three alleles were found significantly less frequently in affected dogs than in control dogs; DLA‐DQB1*00101, DLA‐DQA1*00901, and a *DLA‐DRB1*allele that aligned with a sequence referred to as “*DRB‐T*” in GenBank (Table 2; Figure 2). One of the haplotypes that contained the *DRB‐T* allele (DLA‐DRB‐T/DQA*00101/DQB‐DQBC1) was also found significantly less commonly in affected animals (10.3%) than in control animals (22.0%; OR = 0.41; CI = 0.17‐0.99; *P* = 0.0466; Table 3; Figure 3). Finally, significantly more affected dogs (29.4%) than control dogs (12.1%) were homozygous at all three locations (OR = 3.0; CI = 1.1‐8.6; *P = *0.038).

## 4 DISCUSSION

In this study, we identified four alleles and two haplotypes across three DLA class II genes (*DLA‐ DRB1, DLA‐DQA1, and DLA‐DQB1*) that demonstrated statistically significant association with SARDS in Dachshunds. The region of interest on CFA12 was initially identified through a GWAS, although the association was not statistically significant. However, as the MHC is one of the most highly polymorphic regions of the genome, a GWAS is very unlikely to identify significance of an association in this region.^12^ Therefore, three DLA class II genes were sequenced in a larger population of Dachshunds so as to better identify a true association.

In the present study, the allele DLA‐DRB1*09401and its parent haplotype DLA‐DRB1*09401/DQA*00101/DQB‐DQBC1 were overrepresented in affected dogs, suggesting a higher risk of SARDS in dogs possessing this allele. However, this haplotype also appeared in control dogs, and only about one third of affected dogs possessed at least one copy of the allele. Therefore, this is inconsistent with a single causative mutation for the disease. Rather, it is possible that this allele confers higher risk of developing SARDS, or is associated with this syndrome for some other reason. By contrast, the alleles DLA‐DQB1*00101, DLA‐DQA1*00901, and *DLA‐DRB‐T*, as well as the haplotype DLA‐DRB‐T/DQA*00101/DQB‐DQBC1, were under‐represented in affected dogs, suggesting a protective effect. Again, these potentially protective alleles were found in some SARDS‐affected dogs, and not all control dogs possessed one of the protective alleles. Even when evaluated in combination with the risk allele, there were SARDS‐affected dogs that possessed the protective allele and not the risk allele, as well as control dogs that possessed the risk allele and not the protective allele. These findings reinforce that DLA type does not appear to be solely responsible for susceptibility to developing SARDS.

Associations between MHC genes and autoimmune diseases have been reported in humans^13^, ^14^, ^15^ as well as animals.^16^, ^17^, ^18^ In one study in Doberman pinschers, two of the same DLA alleles suggested by our study to be protective for development of SARDS (DLA‐DQA1*00901 and DLA‐DQB1*00101) were also potentially protective against Doberman hepatitis.^19^ Like SARDS, Doberman hepatitis is a poorly understood disease seen more commonly in female dogs and hypothesized to have an autoimmune etiology. Despite the identification of some novel genetic associations in the present study, the mechanism by which variations in the MHC might predispose an animal or human to autoimmune disease remains unknown. It has been hypothesized that the role of the MHC in antigen presentation to T lymphocytes during development of immunologic tolerance may be involved,^20^, ^21^, ^22^ but a precise mechanism has not been identified.

As the etiology of SARDS is not yet understood, identification of a genetic association between this disease and some DLAs that bears close resemblance to associations found between the MHC and a number of autoimmune diseases is important and supports further investigation of an autoimmune etiology for SARDS. However, spurious associations are often found when genotyping the DLA in this manner, especially in dog breeds with relatively few haplotypes.^23^ However, our study identified twenty‐five different haplotypes in the population studied, making Dachshunds a more genetically diverse breed than some of those previously studied,^24^, ^25^ and lowering the likelihood that this exerted an unwanted effect. Likewise, while the MHC may play a role in SARDS, other genetic and/or environmental factors are likely involved. In order to identify other genetic factors, a larger number of SARDS‐affected and control dogs should be assessed in a GWAS. The GWAS performed here was not subject to significant population stratification, and a large number of SNPs were used; however, it had a modest number of samples more appropriate for the identification of a simple recessive locus.^26^

In conclusion, we have identified several alleles and haplotypes of DLA class II genes which appear to confer either susceptibility to or protection against development of SARDS in the Dachshund. If these are true associations, their presence supports the hypothesis that SARDS has an autoimmune etiology, and this warrants future research.
